# The influence of oxygen tension on the structure and function of isolated liver sinusoidal endothelial cells

**DOI:** 10.1186/1476-5926-7-4

**Published:** 2008-05-05

**Authors:** Inigo Martinez, Geir I Nedredal, Cristina I Øie, Alessandra Warren, Oddmund Johansen, David G Le Couteur, Baard Smedsrød

**Affiliations:** 1 Department of Cell Biology and Histology, IMB, Department of Medicine, IKM, Department of Orthopaedic Surgery, IKM, University of Tromsø, Norway; 2 Surgical Research Lab, IKM, University of Tromsø, Norway; 3 Centre for Education and Research on Ageing and the ANZAC Research Institute, Concord RG Hospital and University of Sydney, Australia

## Abstract

**Background:**

Liver sinusoidal endothelial cells (LSECs) are specialized scavenger cells, with crucial roles in maintaining hepatic and systemic homeostasis. Under normal physiological conditions, the oxygen tension encountered in the hepatic sinusoids is in general considerably lower than the oxygen tension in the air; therefore, cultivation of freshly isolated LSECs under more physiologic conditions with regard to oxygen would expect to improve cell survival, structure and function. In this study LSECs were isolated from rats and cultured under either 5% (normoxic) or 20% (hyperoxic) oxygen tensions, and several morpho-functional features were compared.

**Results:**

Cultivation of LSECs under normoxia, as opposed to hyperoxia improved the survival of LSECs and scavenger receptor-mediated endocytic activity, reduced the production of the pro-inflammatory mediator, interleukin-6 and increased the production of the anti-inflammatory cytokine, interleukin-10. On the other hand, fenestration, a characteristic feature of LSECs disappeared gradually at the same rate regardless of the oxygen tension. Expression of the cell-adhesion molecule, ICAM-1 at the cell surface was slightly more elevated in cells maintained at hyperoxia. Under normoxia, endogenous generation of hydrogen peroxide was drastically reduced whereas the production of nitric oxide was unaltered. Culture decline in high oxygen-treated cultures was abrogated by administration of catalase, indicating that the toxic effects observed in high oxygen environments is largely caused by endogenous production of hydrogen peroxide.

**Conclusion:**

Viability, structure and many of the essential functional characteristics of isolated LSECs are clearly better preserved when the cultures are maintained under more physiologic oxygen levels. Endogenous production of hydrogen peroxide is to a large extent responsible for the toxic effects observed in high oxygen environments.

## Background

The liver sinusoids are lined by endothelial cells that have a unique structure and function essential for hepatic and systemic homeostasis. Much of our understanding of the biology of the LSECs has been generated in experiments curried out on cultured LSECs, mostly derived from rodents. However, *in vitro *preservation of functionally intact LSECs during isolation and culture has been a challenge because isolated LSECs have poor viability and rapidly loose many of their functional and morphological characteristics [1,2]. Some improvements have been achieved with autologous serum [3], hepatocyte-conditioned medium [2], VEGF or sophisticated synthetic serum-free medium [4].

Traditionally, most of the cell cultivation of today is performed in static culture systems maintained under atmospheric or hyperoxic oxygen levels (20%). As yet, the effects of different oxygen tensions on isolated LSECs have not been investigated. Oxygen is an important modulator of cellular function in both normal and disease states. Thus, hypoxic conditions (5–15 mmHg O_2_) are characterized by a shift to more anaerobic metabolic processes in the cells, or to the expression of signalling molecules that promote oxygen delivery, such as pro-angiogenic switches [5]. In contrast, hyperoxic conditions (≥ 160 mmHg O_2_) often results in the formation of reactive oxygen species that are directly implicated in the induction of cell injury via lipid peroxidation and expression of pro-inflammatory cytokines [6]. In the liver, baseline metabolism and functions occur typically in normoxic environments ranging from 30–90 mmHg O_2_. Thus although oxygen gradients occurs between the periportal and perivenous parts of the liver lobule, average oxygen tension is always significantly lower than atmospheric oxygen tension (160 mmHg O_2_). Variation in oxygen levels could represent a critical element in LSEC viability because it drastically interferes with cellular energy metabolism and the generation of oxidative stress. LSECs are particularly sensitive to hyperoxia and oxidative stress induced either by hydrogen peroxide or tert-butylhydroperoxide [7,8]. Accordingly strategies to reduce oxidative stress such as lowering the oxygen tension might be useful in preserving funtional LSECs.

In this study we compared essential morphological and functional features of LSECs during *in vitro *culture using either atmospheric oxygen tension or more reduced oxygen conditions. The results indicate that most LSEC functions are better preserved when the cells are incubated under low oxygen tension.

## Results

### In vivo and in vitro oxygen tension

Baseline oxygen levels were measured in blood samples from the portal vein, hepatic artery or the hepatic vein of anesthetized animals kept mechanically ventilated to stabilize body constants. Similarly, baseline measurements in culture supernatants were obtained after 24 h in CO_2 _incubators adjusted to either 20% O_2 _or 5% O_2_. Results in Table 1 show absolute values of oxygen measurements given in kilo Pascals (kPa). Of note, oxygen levels encountered in cultures maintained at 5% O_2 _are slightly higher than the values found in venous blood entering and leaving the liver.

**Table 1:** In vivo and in vitro oxygen measurements.

	***In vivo***			***In vitro***	
		
	**Portal vein**	**Hepatic vein**	**Hepatic artery**	**5%CO**_2_**/95% air**	**5%CO**_2_**/5%O**_2_
		
**Oxygen tension (kPa)**	7.22 (± 0.9)	6.70 (± 0.3)	20.9 (± 4.6)	18.46 (± 1.2)	7.39 (± 0.9)

Blood samples were collected from the indicated vascular beds and from 24 h-conditioned culture supernatants. Glass capillary tubes were used to avoid equilibration of the samples with atmospheric oxygen. The total oxygen content in the samples is given in kilo Pascals (kPa). The results are representative data obtained from three independent measurements.

### Cell viability assays and morphological analysis

**Figure 1:**
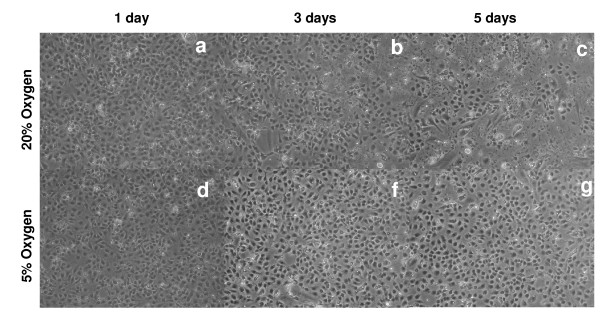
**Morphological examination of LSEC cultures over time by light microscopy**. Freshly isolated LSECs cultures were established on 24 well plates and incubated either at hyperoxia (a-c) or at normoxia (d-f). The general morphology of the cultures was monitored by light microscopy at day 1 (a, d), day 3 (b, d) and day 5 (c, f) after isolation. Decline of LSECs cultures may be observed in dishes maintained at atmospheric oxygen levels (a-c) after several days of culture.

**Figure 2:**
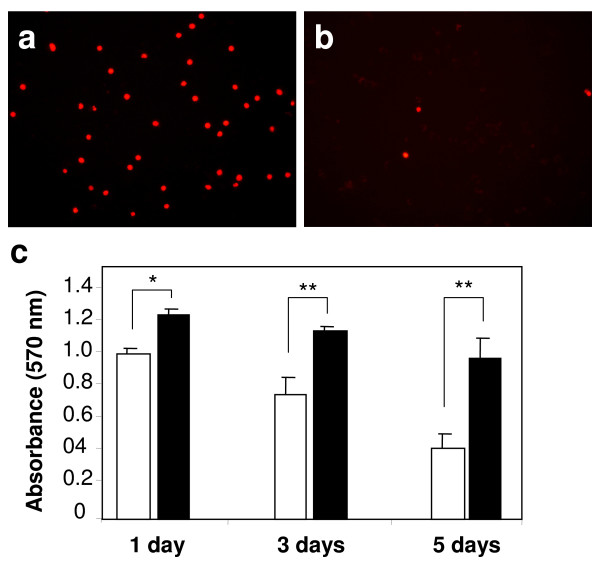
**Comparative viability of LSECs cultures maintained under high and low oxygen levels**. Cell death was monitored by incorporation of propidium iodide in late apoptotic or necrotic cells in cultures maintained in either high (a) or low (b) oxygen tension. Separately, viability was determined at the indicated time points by MTT colorimetric assay (c). Freshly isolated LSECs cultures were established on 24 well-plates and incubated either at hyperoxia (open bars) or at normoxia (filled bars). The obtained results demonstrate a faster decay of loss of cells in cultures maintained at hyperoxic conditions. Statistical analyses by t-student test: *P < 0.05, **P < 0.001.

All *in vitro *experiments in this study were carried out with an especially tailored serum-free medium which has been shown to preserve LSECs morphology and viability better than regular RPMI, DMEM or their serum-containing variants. Quickly after isolation the cells were placed in atmospheric or low oxygen environments and the morphologic development of the culture was monitored over time by conventional light microscopy. LSEC proliferation analysed by BrDU incorporation was undetectable at any culture condition (data not shown). No significant differences in the morphology were observed during the first 48 h of culture (Fig. 1a, 1d). However, the number of viable cells per well, as measured by the MTT assay significantly decreased at hyperoxic conditions already at 24 h (Fig. 2). From the 3^rd ^day in culture, LSECs maintained at atmospheric oxygen tension started to collapse gradually, as observed by the formation of small areas with rounded dying cells and detached cells scattered all over the cultures (Fig. 1b, 1f). The areas with dead cells and cell-remnants were more prominent at the 5^th ^day of cultures kept at high oxygen levels, representing about 85% of the total seeded area, whereas the LSEC cultures maintained at 5% oxygen preserved intact morphology at the end of the experiment (Fig. 1c, 1g). The MTT measurements also confirmed the faster decay of LSEC cultures incubated at high oxygen levels (Fig. 2c). Necrotic and late apoptotic cells detected by incorporation of propidium iodide were more abundant in cultures maintained at high oxygen tension three days after isolation (Fig. 2a, 2b).

### Scanning electron microscopy

**Figure 3:**
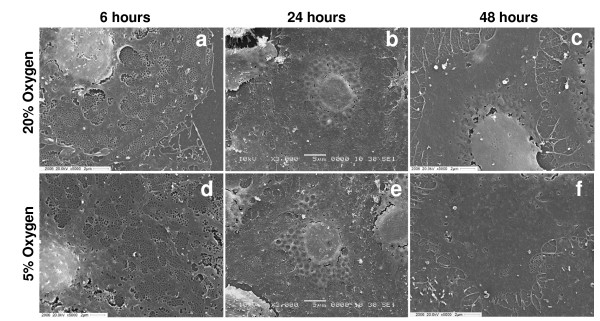
**Fenestration pattern in cultured LSEC analysed by scanning electron microscopy (SEM)**. The evolution of fenestrae in isolated LSECs seeded on fibronectin-coated coverslips was monitored at different time points by SEM. LSECs cultures were maintained at high (a-c) or low (d-f) oxygen levels. Highly fenestrated cells can be observed during early time points of culture. Fenestration is gradually lost over time in both normoxic or hypoxic conditions.

**Figure 4:**
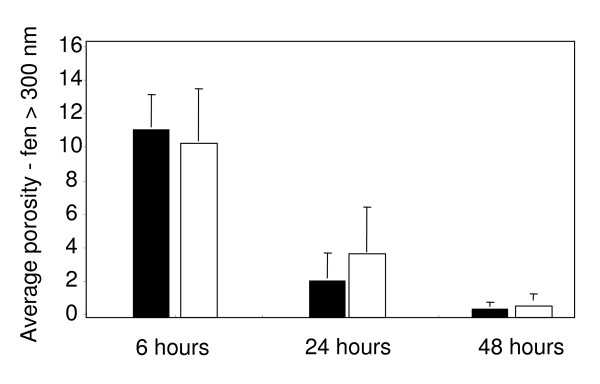
**Porosity measurements**. Porosity analysis of LSECs seeded on coverslips at 6, 24 and 48 h. Fenestrae and gaps greater that 300 nm were excluded from the analysis. Porosity measurements are expressed as percentage of the total area covered by cells in each coverslip. Black columns: 20% oxygen. White columns: 5% oxygen.

Fenestrations represent a specific morphological feature of LSECs. During the initial hours of culture, LSECs demonstrated a well differentiated fenestration pattern with large numbers of fenestrations clustered into liver sieve plates (Fig. 3a–c). However fenestration was drastically reduced after day 1 and practically disappeared after day 2, regardless of the oxygen tension (Fig. 3d–f). Average porosity of cells calculated by direct counting showed that fenestration is rapidly lost in plated LSECs independently of the oxygen levels (Fig. 4). Interestingly, fenestrations appeared to be better preserved in LSECs seeded on collagen-coated dishes than on fibronectin-coated dishes (data not shown).

### Scavenger receptor-mediated endocytosis

**Figure 5:**
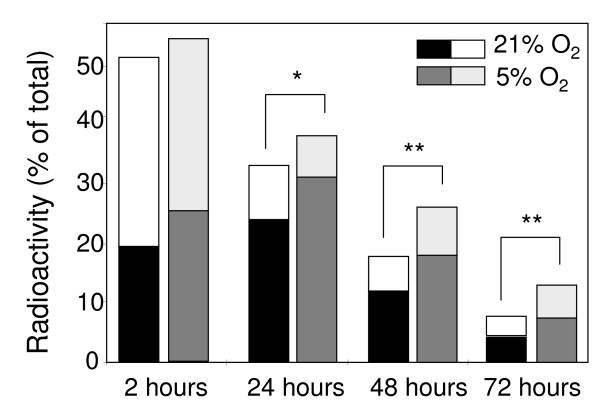
**Time-course analysis of LSECs endocytic capacity**. Endocytosis of ^125^I-FSA, a ligand for the LSEC scavenger receptor was monitored at different time points after incubation of cells at normoxic or hyperoxic conditions. Each column represents separate values of cell-associated (lower part) and degraded (upper part) ^125^I-FSA. Total endocytosis is the result of adding cell associated and degraded ligand (full column size), calculated as percentage of total ^125^I-FSA added to cultures. Values are means of triplicate measurements. The results are representative data obtained from three independent experiments. Statistical analyses by Student's *t*-test: *P < 0.05, **P < 0.001.

Under hyperoxia the endocytic capacity was reduced by approximately 50% within 24 h compared with freshly isolated cultures, and had decreased by about 75% by day 2 and 90% by day 3 (Fig. 5). Under normoxia, the loss of endocytic activity was attenuated, being reduced by 32% at day 1, 65% at day 2 and 75% by day 3 (Fig. 5). Of note, the degradation capacity measured in the cultures in terms of acid soluble radioactivity was largely lost within the first 24 h at either oxygen tensions (Fig. 5).

### Expression of ICAM-1

Surface expression of ICAM-1 measured by flow cytometry on LSECs maintained for 24 h at diferent oxygen levels showed slightly higher scores in LSECs incubated at hyperoxia, compared with normoxia. Relative mean fluorescence values were 966 for 20% oxygen treatments and 819 for 5% oxygen treatments (Fig. 7)

**Figure 6:**
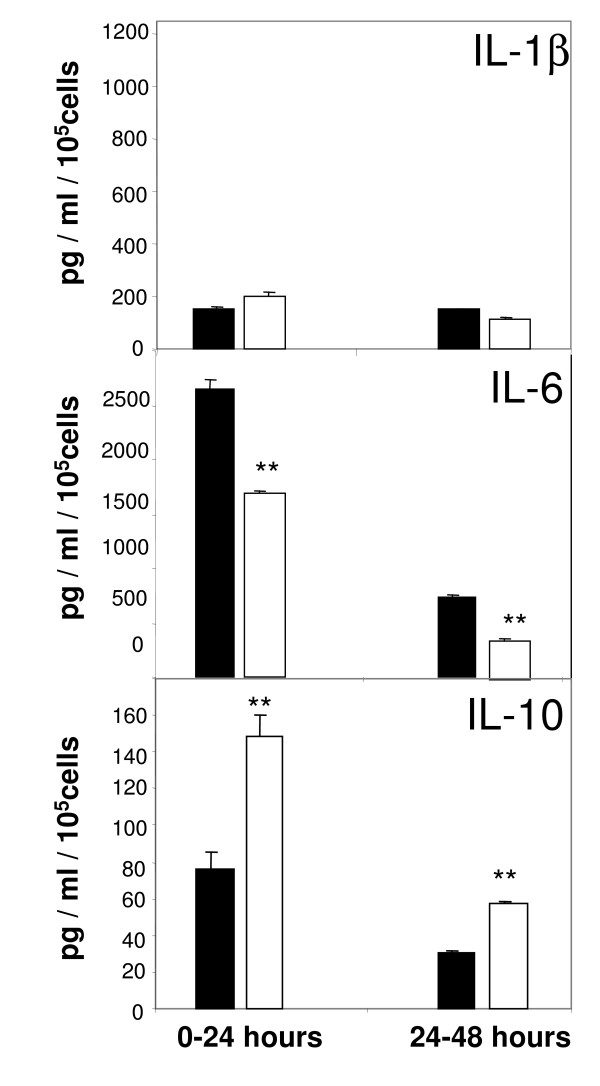
**Production of inflammatory cytokines by LSECs**. Secretion of IL-1β (a), IL-6 (b), or IL-10 (c) was measured in LSEC culture supernatants by ELISA at different time-points. Conditioned media were collected from LSECs cultured at hyperoxia (open bars) or normoxia (filled bars) at 24 hours intervals. Final concentrations were estimated from individual standard curves. Values are means of triplicate measurements. The results are representative data obtained from three independent experiments. Statistical analyses by t-student test: *P < 0.001.

### Lactate production and glucose consumption

Regardless whether LSECs were cultivated under normoxic or hyperoxic conditions, LSECs consumed insignificant amounts of glucose (Table 2). In contrast, LSECs secreted large amounts of lactate to the supernatant. This lactate production was 2.5 times higher at normoxic conditions compared with hyperoxic oxygen levels.

**Table 2:** Lactate and glucose measurements in supernatants obtained from dishes with or without cells.

**Culture Supernatants**	**Time (h)**	**Lactate **(mmol/L/10^6 ^cells)	**Glucose **(mmol/L/10^6 ^cells)
Cell-free (20% O_2_)	0–24	0.10 (± 0.20)	11.81 (± 0.24)
LSECs (20% O_2_)	0–24	1.19 (± 0.42)	10.96 (± 0.40)
LSECs (5% O_2_)	0–24	2.77 (± 0.59)	10.83 (± 0.20)
LSECs (20% O_2_)	24–48	1.31 (± 0.38)	10.70 (± 0.20)
LSECs (5% O_2_)	24–48	3.07 (± 1.12)	10.36 (± 0.20)

Conditioned media collected from cell free incubations, or LSEC cultures were analysed for glucose and lactate. Values are means of triplicate measurements. The results are representative data obtained from three independent experiments.

### Production of inflammatory cytokines

Immunoabsorbent assays performed with cell supernatants revealed that IL-1β is minimally expressed by LSECs and remains the same in both tested oxygen conditions (Fig. 6, upper pannel). IL-1β production by LSEC was induced in control experiments challenged to 10 μg/mL LPS (data not shown). In contrast, endogenous IL-6 is actively released by LSECs cultured under hyperoxic oxygen levels, whereas the levels of this cytokine are reduced by 40% under normoxic conditions during the first 24 h and up to 48 h of culture (Fig. 6, middle panel). Production of the anti-inflammatory cytokine IL-10 correlated inversely with IL-6. The levels of IL-10 measured in supernatants of LSECs cultured under normoxia were twice the levels found in hyperoxia (Fig. 6, bottom panel). This scenario persisted also the following 24 h of culture.

**Figure 7:**
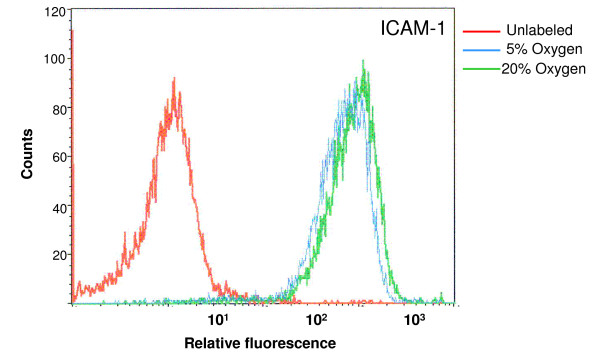
**ICAM expression on cultured LSECs**. Quantitative measurements of ICAM-1 expressed on the surface of LSEC were done by flow cytometry after incubation of LSECs on normoxic and hyperoxic environments during 24 h. Relative mean fluorescence values for 20% O_2 _levels were 966, whereas 5% O_2 _scored 819. The results are representative data obtained from two independent experiments.

### Production of reactive oxygen and nitrogen species

**Figure 8:**
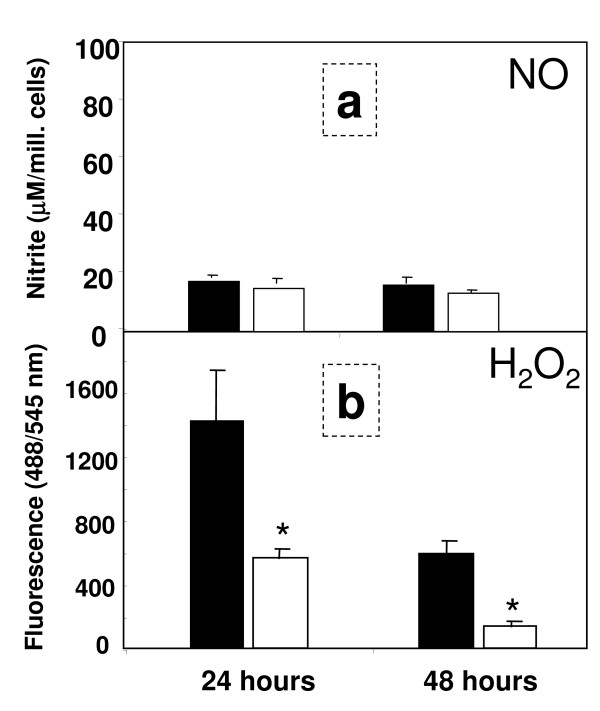
**Production of nitric oxide (NO) and hydrogen peroxide (H_2_O_2_) by LSEC**. Secretion of nitric oxide (NO) was measured in LSEC culture supernatants by Griess reaction at 24 and 48 h (a). Culture media was collected from hyperoxic conditions (open bars) or normoxic conditions (filled bars) at 24 hours intervals. Final concentrations were estimated from individual standard curves. Generation of endogenous H_2_O_2 _was monitored in separate experiments at the indicated time-points in LSEC cultures by H_2_O_2_-mediated oxidation of DCFH-DA into DFC during 6 h (b). Values are total fluorescence emitted at 545 nm.

**Figure 9:**
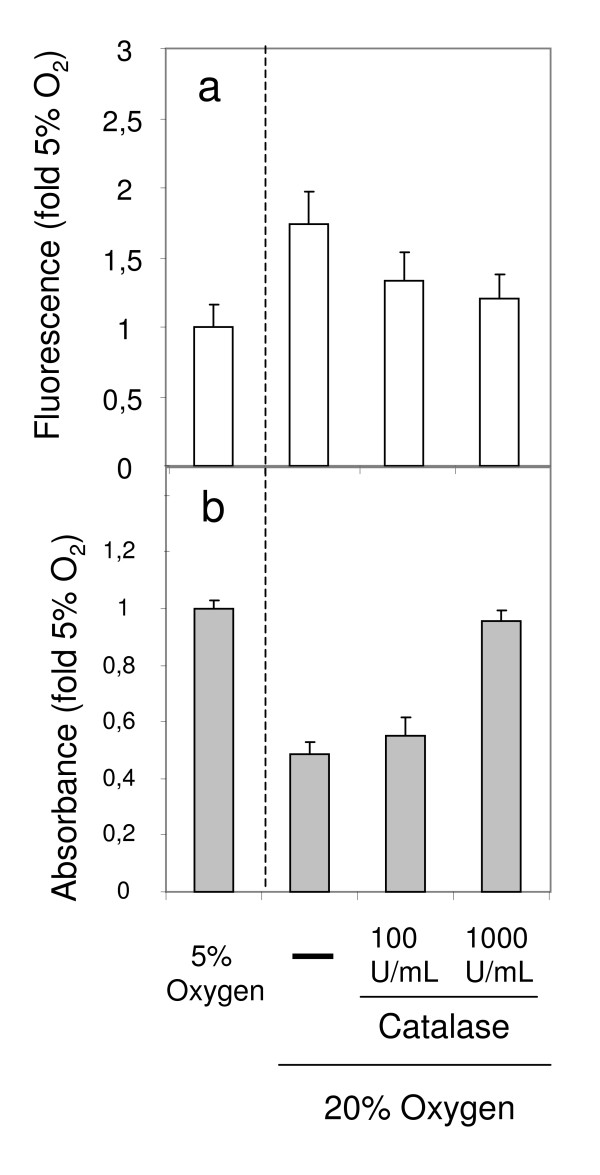
**Effect of catalase on H_2_O_2 _formation and on cell survival in LSEC cultures**. Formation of H_2_O_2 _was measured on LSEC cultures (fluorescence values) during 6 h following the first 24 h of incubation at 5% O_2_, or at 20% O_2 _in the presence or absence of catalase (a). Additionally, survival rates at the third day of culture (absorbance values) were measured on dishes treated the same way (b). All results are representative data obtained from two independent experiments.

There was little expression of NO during the first 48 h of culture at both oxygen tensions (Fig. 8a). Elevation of NO levels was observed in LPS-treated cultures that were used as positive controls (data not shown). LSECs cultured at hyperoxia generated nearly three – fold larger amounts of H_2_O_2 _when compared to LSECs maintained at normoxia (Fig. 8b). The levels of H_2_O_2 _approached the assay detection limit at 48 h of culture in LSECs kept at lower oxygen. Exogenous administration of 1000 U/mL of catalase from the beginning of cell culture, efficiently blocked the production of endogenous H_2_O_2 _during the first 48 hours of culture (Fig. 9a) and was able to revert the cell survival rates observed in cultures kept at hyperoxic conditions (Fig. 9b).

## Discussion

In this study, we demonstrate that atmospheric oxygen levels represent a deleterious environment for LSECs, and that different oxygen environments induce significant functional and structural changes in these cells. LSECs are known to be particularly vulnerable to variations of oxygen levels, as demonstrated by ischemia-reperfusion challenges of livers, or in anoxia-reoxygenation experiments *in vitro *[9]. During the re-oxygenation periods LSECs, KCs and hepatocytes produce large amounts of oxygen radicals, and LSECs gradually go into apoptosis induced by oxidative stress [8]. These findings suggest that LSECs are poorly equipped to adapt to hyperoxic conditions. It is therefore a curious fact that all published studies using cultured LSECs have been conducted with cells maintained under atmospheric or hyperoxic oxygen tension. We here demonstrate that LSECs cultured under moderately low oxygen tension exhibit improved survival and maintain their *in vivo *characteristics better as compared to LSECs cultured under atmospheric oxygen tension.

At physiologic conditions the ratio of portal vs. arterial blood flow entering the liver is around 4:1. In the rat system, most arterial blood reaches the sinusoids indirectly, via initial anastomosis between the terminal hepatic arteriole and the portal venule [10]. In average, the oxygen content of hepatic blood is rather low (~55 mmHg). However, oxygen gradients normally exist between the periportal and the perivenous areas of the liver lobule, ranging from 60–70 mmHg O_2 _in the periportal area to 25–35 mmHg O_2 _in the perivenous areas [11]. Some laboratories have developed complex bioreactor systems that allow the formation of steady state oxygen gradients in culture [12]. Zonal heterogeneity with regard to the oxygen tension has been studied in hepatocyte cultures but not in cultured LSECs. Practically all published *in vitro *studies on LSECs are conducted in static culture systems where the levels of oxygen have not been an issue.

Many research groups use long-term cultures of LSECs to explore these cells [13,14]. In spite of this fact, surprisingly few studies have focused on identifying changes induced on LSEC functions, morphology or viability during normal *in vitro *culture. Nevertheless, some laboratories have reported that signature LSEC functions such as endocytic capacity or fenestration are drastically reduced after 1 or 2 days in culture [4,1]. Some research groups have attempted to develop protocols to extend the *in vitro *lifetime of functionally intact LSECs. Most strategies have used specially designed culture media, including tumour-conditioned medium [15], hepatocyte-conditioned medium [2], autologous rat serum [3], phorbol ester supplemented medium, vascular endothelial growth factor-containing medium [16] or orthovanadate [1]. In the present study we used a serum-free medium developed previously in our laboratory [4]. With this medium, we were able to maintain fuctionally intact rat LSEC cultures during 48 h. However, from the third day of incubation, the cultures gradually lost their integrity. This loss was nevertheless prevented by incubation of cultures under low oxygen tension, enabling maintenance of intact morphology after six days of culture. This demonstrates that atmospheric oxygen levels itself is a disfavourable environment for LSECs.

Several reports have shown that a major biological function of LSECs is to rid the blood of an array of naturally occurring soluble macromolecular and colloidal waste substances, via clathrin-mediated endocytosis (for review see [17]). Based on the knowledge that receptor-mediated endocytosis represents a characteristic function of LSECs, we compared the ability of the cells to internalize and degrade formaldehyde treated serum albumin (FSA), that is specifically taken up by the scavenger receptor of LSEC, in high and low oxygen environments. Although the endocytic capacity decreased gradually over time in both conditions, the total uptake measured at 24, 48 and 72 h was significantly higher under physiological oxygen conditions than under hyperoxic conditions. Yet, culturing of LSECs under low oxygen levels per se was not enough to maintain the endocytic capacity at the same level as measured in freshly prepared cultures. The reason for this is at least three-fold, based on the fact that LSEC monocultures lack: i) essential factors produced locally or brought to the cells via the portal circulation, ii) interaction with other liver cells, and/or iii) interaction with native extracellular matrix. Focusing in the present work on the impact of oxygen tension, we show here that a low oxygen level *in vitro*, approaching that of the local sinusoidal environment *in vivo*, significantly prolong the naturally high scavenger activity of LSECs when compare to traditional cultures established at atmospheric oxygen levels.

Another important physiological function carried out by LSECs *in vivo *is the filtration of numerous plasma components towards the liver parenchyma through a well organized net of transcytoplasmic holes called fenestrae. This fenestration represents a hallmark of intact mammalian LSEC. It is noteworthy that several reports conclude that the cells undergo a rapid defenestration after isolation and culture [18]. Assessing fenestration using scanning electron microscopy, we found that this feature is gradually lost over time independent of the culture conditions used. Of note, LSECs lost fenestration more rapidly when seeded on fibronectin-coated dishes than on collagen-coated ones. This shows that the key factors which enable the maintenance of the LSEC fenestrae in the intact liver are lost upon cultivation, regardless the oxygen levels.

Adaptation to hypoxia is known to induce changes in the energy metabolic routes of cells, most commonly shifting from the oxidative phosphorylation pathways to the glycolytic routes. Morphometric studies of LSECs in the intact liver have shown that the cells contain unusually few mitochondria [19,20]. This observation, along with the fact that rat LSECs produce large amounts of lactate and acetate, even when cultured at high oxygen levels, strongly suggest that these cells are geared to a largely anaerobic type of metabolism. Conceivingly, LSECs perform less oxidative phosphorylation compared to most other cells types, and it has been suggested that in LSECs, glutamine and fatty acid oxidation are the main sources of energy [21]. An alternative path is the anaerobic conversion of pyruvate, originating from the catabolism of glucogenic aminoacids from the growth medium, into lactate [22]. Examining glucose consumption and lactate production under different oxygen tensions to explore possible variations in the energy sources of the cells, we found that glucose was not consumed by LSECs under hyperoxic or normoxic conditions. This suggests that the energy metabolic routes are independent of the oxygen levels. In contrast, the amount of lactate generated by LSECs under normoxic conditions was enhanced almost three times, suggesting that metabolic energy reactions are driven more efficiently under low oxygen.

As a rule, procedures used to isolate and cultivate cells induce cell activation to some extent. Desirable *in vitro *models should be based on non-activated or low-activated cells. In the present study we measured the production of inflammatory cytokines, reactive oxygen species and the expression of adhesion molecules after cell cultivation as indicators of cell activation. Our results confirm that LSECs produce high levels of IL-6 when cultured under "standard" high (20%) oxygen pressure. Notably, the expression of this cytokine was reduced by 50% when the cells were maintained at low oxygen levels. In contrast, the production of IL-10, an anti-inflammatory mediator, was enhanced when the cells were incubated at 5% oxygen. Flow cytometric analysis of ICAM-1 expression at the cell surface show strong signal on LSECs cultured for 24 h at 20% oxygen. These values are however slightly reduced upon incubation of cells at low oxygen tensions. The overall results indicate that LSECs have a less activated phenotype when they are incubated at low oxygen levels.

The transfer of LSECs from an *in vivo *low oxygen tension to an *in vitro *high oxygen tension may exert effects on the cells similar to those observed in LSECs during hypoxia-reoxygenation of the intact liver. Indeed, we observed large production of hydrogen peroxide by LSECs when the cells were kept at atmospheric oxygen conditions. Of note, this production was much lower at low oxygen tension. Hydrogen peroxide induces toxic effects on LSECs, mostly because these cells are not well equipped to metabolize this reactive substance [8,9]. Addition of catalase to the medium, an enzyme that mediates directly the catabolism of H_2_O_2_, was able to abrogate to a large extent the formation of H_2_O_2_, and had beneficial effects on the morphology and survival of LSEC cultures. We thus entertain the idea that endogenous H_2_O_2 _may be largely responsible for the rapid culture decline observed during high oxygen incubation.

## Conclusion

In this study we report that atmospheric oxygen tension has harmful effects on isolated rat LSECs during long term cultivation. These effects may be largely abrogated by incubation of the cells at physiological O_2 _conditions. Our findings are compatible with a better preservation of essential morphologic and functional LSEC features under more physiological oxygen tensions. Based on these data, we recommend low oxygen environments for cultivation of LSECs, especially when long-term cultures are used.

## Methods

### Isolation and culture of LSEC

Preparation of highly purified rat liver sinusoidal endothelial cells was performed as previously described [23]. Briefly, rat livers were perfused with collagenase (Worthington Biochemical Corporation, Lakewood, NJ, USA.) and the resulting suspension of single cells was subjected to low speed centrifugation to eliminate most of the hepatocytes, followed by discontinuous density centrifugation in Percoll (Amersham Biotech, Uppsala, Sweden) gradients. The resulting non-parenchymal cells were suspended in pre-warmed serum-free culture medium. Kupffer cells were eliminated by selective attachment to glutaraldehyde-treated albumin (Ostapharma, Ziegelbrucke, Switzerland), and the enriched LSEC suspension was seeded on dishes coated with either rat tail collagen type I (Nutacon, Leimunden, Netherlands) or human fibronectin (isolated at the laboratory from pooled human blood samples). For all experiments the cells were incubated in DM110/SS serum-free medium [4]. Incubations were undertaken under hyperoxia (20%) or normoxia 95%N_2_/5%O_2 _to reflect physiologic conditions in the liver sinusoids. The monolayer cultures were monitored by conventional light microscopy.

### Endocytosis measurements

Cultures of LSEC (0.5 × 10^6^) were established in 2 cm^2 ^wells and maintained in serum-free medium. After experimental treatments, cells were cultured for 90 min at 37°C in 250 μl of RPMI, containing 1% Human Serum Albumin (HAS) and trace amounts (40.000 cpm, 50 ng/ml) of radioiodinated formaldehyde-treated bovine serum albumin (^125^I-FSA). Endocytosis experiments were terminated by transferring incubation medium and two washing volumes to tubes containing 800 μl of 20% TCA, thereby inducing precipitation of non-degraded proteins. After centrifugation, precipitated and soluble radioactivity was measured using a Geiger counter. Acid soluble radioactivity was considered to indicate degraded FSA, and results were evaluated from total radioactivity added to cultures. Cell associated radioactivity was calculated by solubilisation of cell monolayers with 1% SDS, and measured with a γ-counter (Cobra II, Packard). Values were normalized for the total number of cells counted per well.

### Scanning electron microscopy

Scanning EM was performed as previously described [24]. LSECs that had been cultured in hypoxic and normoxic conditions for 6, 24 and 48 h were fixed for 1 hour with 2.5% glutaraldehyde in 0.1 mol/l sodium cacodylate buffer (1% sucrose). Coverslips were treated with tannic acid (1% in 0.15 mol/l cac. buffer), osmicated (1% OsO4/0.1 mol/l cacodylate buffer), dehydrated in a series of ethanol gradients and finally incubated in hexamethyldisilazane for 2 min. Gold coated coverslips were viewed using a Jeol scanning microscope. Five representative cells from each time point were photographed (magnification 3,000–5,000 ×) and fenestral diameter and porosity (percentage of surface area occupied by fenestrations) analysed using ImageJ software. The results are expressed as mean ± S.D.

### ELISA measurements of cytokine production

The production of the inflammatory mediators IL-1β, IL-6 and IL-10 in LSEC culture supernatants was determined with specific rat IL-1β, IL-6 and IL-10 ELISA kit (R&D Systems, Minneapolis, USA) according to manufacturers' instructions. Briefly, after the experimental incubations of cultures, the supernatants were collected and underwent high speed centrifugation, then kept at -20°C. 96 well-plates were coated with "capture" antibodies and 100 μl of 1:2 diluted supernatants were added to each well and incubated for 2 h at room temperature. The "detection" antibody was then applied for 2 h and the wells were ultimately subjected to peroxidase reaction. Absorbance was measured at 450 nm and the values were converted into μg/ml according to the standard curve. Values were normalized after the total number of cells counted per well.

### ICAM expression by flow cytometry

For measurements of ICAM-1 expression at the cell surface, LSEC were incubated at different oxygen tension during 24 h, detached from wells by 20 min incubation in EDTA buffer, and immediately fixed in 4% paraformaldehyde. Fixative was removed by cell sedimentation and the cells were then resuspended in 500 μl of PBS containing 1% BSA. Specific monoclonal antibody against rat ICAM (Biodesign International, Saco, ME, USA) was added to tubes containing fixed LSEC and incubated during 30 min at room temperature. Unlabeled antibody was eliminated by a series of cell washings, followed by incubation with a secondary antibody against mouse IgG FITC-congugated (Dako Denmark A/S). A group of cells incubated only with secondary antibodies was used as negative controls. Fluorescent cells were then analyzed on a BD FACScan flow cytometer

### Measurement of endogenous H_2_O_2 _production

To measure endogenous production of H_2_O_2_, cells were seeded in 24 well plates and incubated for the indicated time points at different oxygen levels. After incubation, cells were incubated with 20 mM/L 2',7'-dichlorofluorescein-diacetate (DCFH-DA) for 30 min at 37°C. This is a non-polar compound that readily diffuses into cells. The cultures were washed twice to eliminate excess amount of reagent and the cultures were further incubated for 6 h at 37°C. Once the acetate groups are cleaved by intracellular esterase, H_2_O_2 _produced by the cells oxidizes DCFH to the fluorescent compound 2',7'-dichlorofluorescein (DCF). In this way, fluorescence intensity is proportional to the amount of H_2_O_2 _generated. DFC fluorescence was recorded using a computerized plate-scanning microfluorimeter (CytoFluor-2350 system, Millipore Co. Bedford, MA) at both 485/22-nm excitation with 530/25-nm emission filter at high sensitivity settings. Non-DCFH-DA-incubated cells were used to subtract basal auto fluorescence.

### Nitric oxide analysis

The stable end product of nitric oxide, nitrite (NO_2_^-^), was measured in culture supernatants by standard colorimetric assay [25]. Briefly, 50 μl aliquots of medium were collected from individual wells and treated with an equal volume of Griess reagent (1% sulphanilamide and 0.1% napthylenediamide dihydrochloride in 2.5% H_3_PO_4_) at room temperature for 10 min. The optical density of the samples was recorded using TiterTek Multiskan at 540 nm. A standard curve using NaNO2 in clean culture medium was used for calculating NO_2_^- ^concentration. All values are means ± S.D. of triplicate measurements for three separate experiments.

### Lactate, glucose and oxygen measurements

Lactate and glucose concentrations were measured with YSI-analyzer (Cobas, Switzerland). Cells were seeded in 24 well-plates and incubated at different oxygen levels. Cell supernatants were collected every 24 h and cleaned from particles and debris by high speed centrifugation. For each measurement, 150 μl of culture supernatant was utilized. The different oxygen tensions in blood in anesthetised pigs were measured in the portal vein, hepatic vein and aorta. The partial pressures of oxygen and pH in the samples (culture supernatants and blood) were analyzed with a blood-gas analyzer (Rapidlab 865, Chiron Diagnostics, UK). The sampling was performed with a glass capillary tube.

### Cell viability assay: MTT assay

LSEC were seeded on 24 well-plates and cultivated for the indicated time-points. Cells were subsequently exposed to 0.25 mg/mL of MTT (3-[4,5-dimethylthiazol-2-yl]-2,5-dephenyl tetrazolium bromide; Sigma-Aldrich) reagent and incubated for 2 h at 37°C. After 2 h, 200 μL of dissolving solution (96,7% Isopropanol/3,3% HCl) was added to each well, followed by incubation for 1 h at 37°C in a rocker plate. The absorbance at 570 nm of each sample well was measured by using an automated plate reader and compared between the groups.

### Propidium iodide staining for cell death

Necrotic or late apoptotic cells were identified on LSEC cultures by propidium iodide incorporation. Adherent LSEC cultures were established on 2 wells thermanox slides (NUNC International, Tokio, Japan), treated with fibronectin. Cultures were maintained at high and low oxygen environments during three days, and the culture media was renewed every 24 h. Propidium iodide staining was performed with Apoptosis/necrosis detection kit from Calbiochem following manufacturer instructions. The specimen were embedded in Dako Fluoromount (Dako, Glostrup, Denmark), and examined in a fluorescence microscope (Zeiss Axiophot, Germany) equipped with a Nikon DS-5MC digital camera.

### Statistics

Results are presented as mean ± S.E.M. and the two experimental conditions compared using the Student's *t*-test with *P *< 0.05 considered significant. In all experiments, the obtained raw data were normalized against to the amount of cells counted in each treatment. For the quantitative measurements done in SEM, comparisons between groups were undertaken using Kruskal-Wallis test with a post hoc Dunn analysis. P value of < 0.05 was considered statistically significant.

## Competing interests

The authors declare that they have no competing interests.

## Authors' contributions

IM and BS conceived of the study. IM carried out most of the experimental work and the writing. AW and DGL participated in the determination of fenestration parameters, statistical analysis and assisted in the writing. CIØ carried out the isolation and characterization of the cells. GIN participated in the oxygen measurements, as well as in the determination of glucose and lactate in culture supernatants. OJ and BS participated in the design of the study, coordination and helped to draft the manuscript.

## References

[B1] Ohi N, Nishikawa Y, Tokairin T, Yamamoto Y, Doi Y, Omori Y, Enomoto K (2006). Maintenance of Bad phosphorylation prevents apoptosis of rat hepatic sinusoidal endothelial cells in vitro and in vivo. Am J Pathol.

[B2] Krause P, Markus PM, Schwartz P, Unthan-Fechner K, Pestel S, Fandrey J, Probst I (2000). Hepatocyte-supported serum-free culture of rat liver sinusoidal endothelial cells. J Hepatol.

[B3] de Leeuw AM, Barelds RJ, de Zanger R, Knook DL (1982). Primary cultures of endothelial cells of the rat liver: a model for ultrastructural and functional studies. Cell Tissue Res.

[B4] Elvevold K, Nedredal GI, Revhaug A, Bertheussen K, Smedsrod B (2005). Long-term preservation of high endocytic activity in primary cultures of pig liver sinusoidal endothelial cells. Eur J Cell Biol.

[B5] Semenza GL (1999). Regulation of mammalian O2 homeostasis by hypoxia-inducible factor 1. Annu Rev Cell Dev Biol.

[B6] D'Angio CT, Finkelstein JN (2000). Oxygen regulation of gene expression: a study in opposites. Mol Genet Metab.

[B7] Cogger VC, Mross PE, Hosie MJ, Ansselin AD, McLean AJ, Le Couteur DG (2001). The effect of acute oxidative stress on the ultrastructure of the perfused rat liver. Pharmacol Toxicol.

[B8] Motoyama S, Minamiya Y, Saito S, Saito R, Matsuzaki I, Abo S, Inaba H, Enomoto K, Kitamura M (1998). Hydrogen peroxide derived from hepatocytes induces sinusoidal endothelial cell apoptosis in perfused hypoxic rat liver. Gastroenterology.

[B9] Samarasinghe DA, Tapner M, Farrell GC (2000). Role of oxidative stress in hypoxia-reoxygenation injury to cultured rat hepatic sinusoidal endothelial cells. Hepatology.

[B10] Yamamoto K, Sherman I, Phillips MJ, Fisher MM (1985). Three-dimensional observations of the hepatic arterial terminations in rat, hamster and human liver by scanning electron microscopy of microvascular casts. Hepatology.

[B11] Allen JW, Bhatia SN (2003). Formation of steady-state oxygen gradients in vitro: application to liver zonation. Biotechnol Bioeng.

[B12] Allen JW, Khetani SR, Bhatia SN (2005). In vitro zonation and toxicity in a hepatocyte bioreactor. Toxicol Sci.

[B13] Rauen U, Elling B, de Groot H (1997). Injury to cultured liver endothelial cells after cold preservation: mediation by reactive oxygen species that are released independently of the known trigger hypoxia/reoxygenation. Free Radic Biol Med.

[B14] Gerlach JC, Zeilinger K, Spatkowski G, Hentschel F, Schnoy N, Kolbeck S, Schindler RK, Neuhaus P (2001). Large-scale isolation of sinusoidal endothelial cells from pig and human liver. J Surg Res.

[B15] Irving MG, Roll FJ, Huang S, Bissell DM (1984). Characterization and culture of sinusoidal endothelium from normal rat liver: lipoprotein uptake and collagen phenotype. Gastroenterology.

[B16] Esser S, Wolburg K, Wolburg H, Breier G, Kurzchalia T, Risau W (1998). Vascular endothelial growth factor induces endothelial fenestrations in vitro. J Cell Biol.

[B17] Smedsrod B, Pertoft H, Gustafson S, Laurent TC (1990). Scavenger functions of the liver endothelial cell. Biochem J.

[B18] DeLeve LD, Wang X, Hu L, McCuskey MK, McCuskey RS (2004). Rat liver sinusoidal endothelial cell phenotype is maintained by paracrine and autocrine regulation. Am J Physiol Gastrointest Liver Physiol.

[B19] Wisse E (1972). An ultrastructural characterization of the endothelial cell in the rat liver sinusoid under normal and various experimental conditions, as a contribution to the distinction between endothelial and Kupffer cells. J Ultrastruct Res.

[B20] Fujii Y, Ohno N, Li Z, Terada N, Baba T, Ohno S (2006). Morphological and histochemical analyses of living mouse livers by new 'cryobiopsy' technique. J Electron Microsc (Tokyo).

[B21] Spolarics Z, Lang CH, Bagby GJ, Spitzer JJ (1991). Glutamine and fatty acid oxidation are the main sources of energy for Kupffer and endothelial cells. Am J Physiol.

[B22] Nedredal GI, Elvevold K, Ytrebo LM, Fuskevag OM, Pettersen I, Bertheussen K, Langbakk B, Smedsrod B, Revhaug A (2007). Significant contribution of liver non-parenchymal cells to metabolism of ammonia and lactate, and cocultivation augments the functions of a bioartificial liver. Am J Physiol Gastrointest Liver Physiol.

[B23] Smedsrod B, Pertoft H, Eggertsen G, Sundstrom C (1985). Functional and morphological characterization of cultures of Kupffer cells and liver endothelial cells prepared by means of density separation in Percoll, and selective substrate adherence. Cell Tissue Res.

[B24] Cogger VC, Muller M, Fraser R, McLean AJ, Khan J, Le Couteur DG (2004). The effects of oxidative stress on the liver sieve. J Hepatol.

[B25] AH D, CF N, DJ S (1988). Release of reactive nitrogen intermediates and reactive oxygen intermediates from mouse peritoneal macrophages. Comparison of activating cytokines and evidence for independent production. J Immunol.

